# Reporting knee meniscal tears: technical aspects, typical pitfalls and how to avoid them

**DOI:** 10.1007/s13244-016-0472-y

**Published:** 2016-02-16

**Authors:** Nicolae V. Bolog, Gustav Andreisek

**Affiliations:** Phoenix Swiss Med, Mittelweg 29, 4142 Munchenstein, Switzerland; Institute for Diagnostic and Interventional Radiology, University Hospital Zurich, University of Zurich, Ramistrasse 100, 8091 Zurich, Switzerland

**Keywords:** Knee, Magnetic resonance imaging, Menisci, Anatomy, Magnetic fields

## Abstract

**Abstract:**

Magnetic resonance imaging (MRI) is the most accurate imaging technique in the diagnosis of meniscal lesions and represents a standard tool in knee evaluation. MRI plays a critical role in influencing the treatment decision and enables information that would obviate unnecessary surgery including diagnostic arthroscopy. An accurate interpretation of the knee depends on several factors, starting with technical aspects including radiofrequency coils, imaging protocol and magnetic field strength. The use of dedicated high-resolution orthopaedic coils with a different number of integrated elements is mandatory in order to ensure high homogeneity of the signal and high-resolution images. The clinical imaging protocol of the knee includes different MRI sequences with high-spatial resolution in all orientations: sagittal, coronal, and axial. Usually, the slice thickness is 3 mm or less, even with standard two-dimensional fast spin echo sequences. A common potential reason for pitfalls and errors of interpretation is the unawareness of the normal tibial attachments and capsular attachment of the menisci. Complete description of meniscal tears implies that the radiologist should be aware of the patterns and the complex classification of the lesions.

***Teaching points*:**

• *Technical factors may influence MRI interpretation*.

• *Unawareness of the normal meniscal anatomy may lead to errors of interpretation*.

• *Description of meniscal tears implies the knowledge of meniscal tear classification*.

## Introduction

Meniscal tears are a common pathology and diagnosis relies on a detailed clinical history and clinical examination, magnetic resonance imaging (MRI), and arthroscopy. Some types of meniscal tears (e.g. horizontal or oblique tears) may not always be related to clinical symptoms, and they are frequently encountered in asymptomatic knees [1]. It has been shown that meniscal tears are exclusively MRI-based in more than one-third of patients [1]. The anatomical distribution of meniscal tears varies between the medial and the lateral meniscus, and knowing this distribution is helpful in assessing the menisci on MRI [2]. Half ofhe meniscal tears involve the medial meniscus, and in 98 % of the cases, the tear is within the posterior horn and the body of the meniscus [3, 4]. However, at the same time, most of the false-positive diagnoses are also located in the posterior horn of the medial meniscus and are represented by apparent longitudinal tears [5]. On the other hand, the diagnosis of the anterior horn tear of the medial meniscus should be made with caution since it represents only 2 % of medial meniscus tears [6, 7]. Lateral meniscal tear distribution is more variable, with 55 % of the cases involving the posterior horn, 29 % the body or the body and the anterior horn, and 16 % the anterior horn alone [3].

MRI diagnosis is based on the presence of linear signal changes that come in contact with the meniscal surfaces, or is based on the shape and size alterations of the meniscus [7–9]. Nevertheless, the presence of signal changes within the meniscus that are not in contact with the meniscal surfaces are no more likely to represent a significant lesion than a meniscus without any internal changes seen on MRI [10].

The MRI diagnosis performance is high (specificity and sensitivity in diagnosing meniscal tears is high, with a sensitivity of 93.3 % and a specificity of 88.4 % for the medial meniscus and a sensitivity of 79.3 % and a specificity of 95.7 % for the lateral meniscus), but a definitive diagnosis of a meniscal tear can be made on MRI in 95 % of cases, with 5 % remaining in which the diagnosis may not be possible [2, 6].

There are several different factors that may influence the diagnoses of meniscal tears, beginning with technical parameters (radiofrequency coils, imaging protocol and magnetic field strength). Another potential reason for errors of interpretation is the unawareness of the normal tibial attachments and capsular attachment of the menisci. Finally, for a complete description of meniscal tears, the radiologist should be aware of the patterns and complex classification of the lesions [2].

The purpose of this article is to review the technical and anatomical causes of typical pitfalls, to describe how to avoid them, and to improve diagnostic confidence in diagnosing meniscal tears.

## Technical aspects

### Radiofrequency (RF) coils

There are limited possibilities to improve image quality for many of the mature MRI techniques in clinical practice today, and, therefore, the importance of radiofrequency coil design should not be underestimated [11]. Several different types of coils may be used for imaging the knee, including receive-only coils with one or multiple elements, and transmit/receive coils.

Flex coils are non-dedicated surface coils and their use results in several limitations, including a small field-of-view and heterogeneity of the signal intensity through the entire joint. Therefore, nowadays, the flex coils are not indicated for meniscal evaluation.

**Fig. 1:**
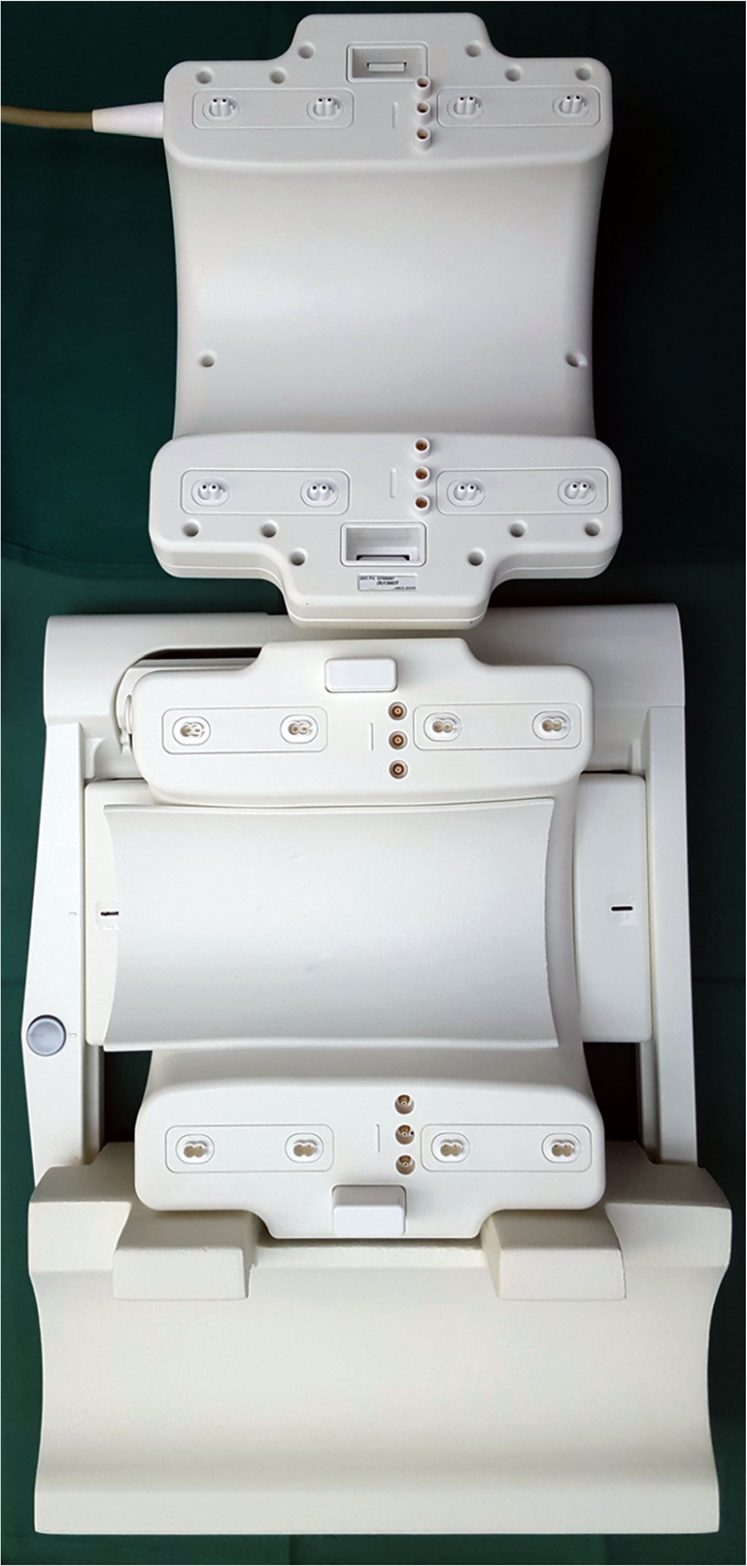
Photograph of an opened, commercially available, dedicated 18-channel transmit / receive knee coil (Quality Electrodynamics, Mayfield Village, OH) for a clinical 3.0 T MR system (Siemens Healthcare, Erlangen Germany)

**Fig. 2:**
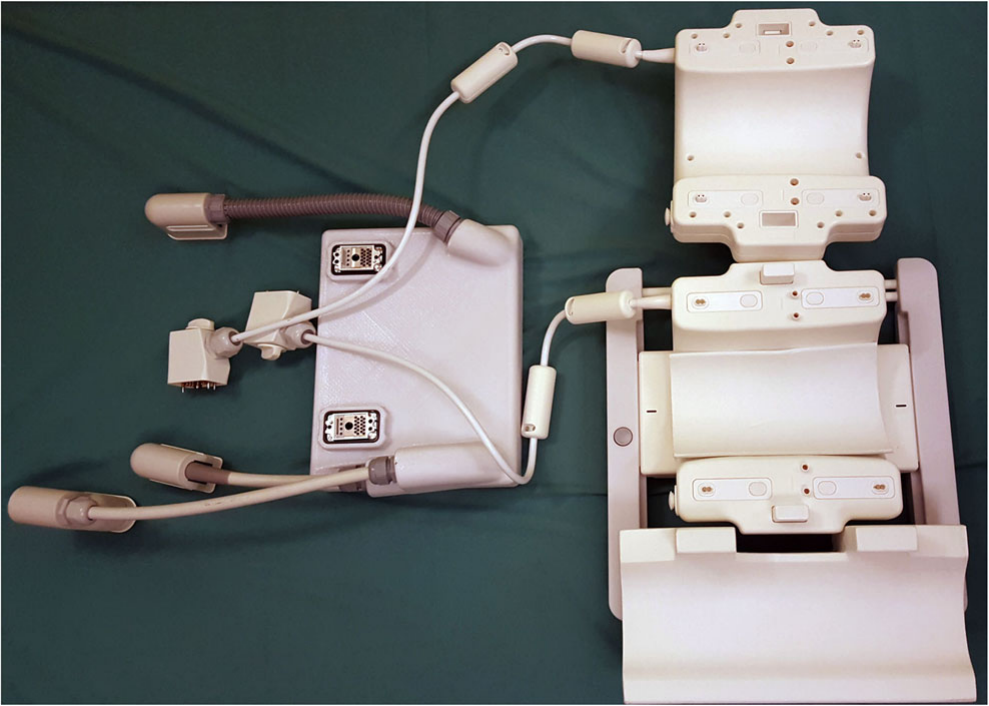
Photograph of an opened, 28-channel transmit / receive knee coil (Quality Electrodynamics, Mayfield Village, OH) for a 7.0 T MR system (Philips, Best, the Netherlands) that includes the coil as well as a special adapter box (left) that connects the coil to the MR system

Quadrature coils or circularly polarized coils can be used as both transmit and receive coils and enable an optimal signal-to-noise ratio through the entire knee joint. The use of these dedicated high-resolution orthopaedic coils with different numbers of integrated elements is mandatory in order to ensure high homogeneity of the signal and high-resolution images (Fig. 1). As higher magnetic field systems (3.0 T, 4.0 T and 7.0 T) are used more and more often in clinical settings and research areas, the limits of performance in pulse sequence acquisition efficiency is approaching the limits [11]. Optimization of RF coils is seen as one of the solutions for further improvement [11]. Recently, dedicated 28-element RF coils with high acceleration factors have been developed for imaging at 7.0 T with very high-resolution (Fig. 2) [12]. However, all these developments imply significant costs and the decision makers must consider the benefits of the investment based on an analysis of effectiveness in relation to cost. The authors, however, strongly recommend the use of dedicated knee coils.

### Protocol considerations

**Fig. 3:**
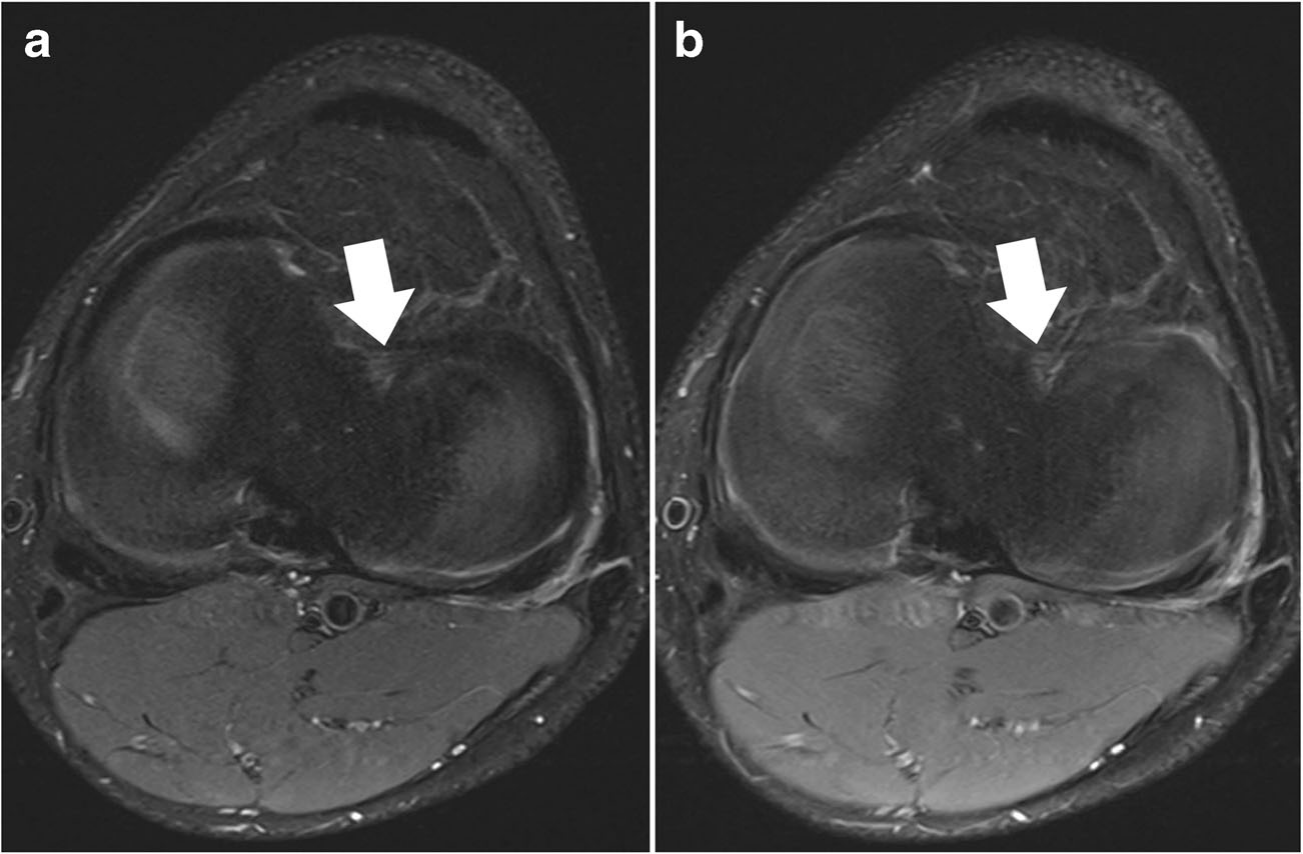
Set of two axial proton-density–weighted fat-suppressed fast spin echo images in a 42-year-old female patient, acquired at 2.5 mm slice thickness (**a**) and 5 mm slice thickness (**b**). With thinner slices, the meniscal root ligaments of the anterior horn of the medial meniscus (*arrows*) are much better appreciated. With thicker slices, these ligaments are barely visible, mainly due to partial volume effects

The clinical imaging protocol of the knee includes different MRI sequences with high-spatial resolution in all orientations: sagittal, coronal, and axial (Table 1). The spatial resolution should be maximized by using small field-of-view, thin slices, and high matrix size. The recommended field-of-view is 16 cm, but it also depends on the joint size and on the type of the radiofrequency coil used. Usually, the slice thickness is 3 mm or less, even with standard two-dimensional fast spin echo sequences. For axial images, the thinner the slice thickness, the better possible evaluation of the meniscal roots (Fig. 3). However, the increase in resolution decreases the signal-to-noise ratio. Although this is a limiting factor, it usually may not be a problem when dedicated knee coils are used at high magnetic field strengths (3.0 T and 7.0 T). Most clinical MR scanners on the market (1.5–3.0 T) provide excellent image quality even when high resolution images (in-plane and through-plane) are the desired goal.

**Table 1:** Example for an MR imaging protocol of the knee

Orientation	Sequence type	Repetition time / echo time [ms]	Fat suppressed	Resolution (mm)	Acquisition time [ms]
sagittal	proton-densitiy turbo spin echo	3000 / 29	No	0.2. × 0.2 × 2.8	3:35
sagittal	T2-weighted turbo spin echo	4600 / 83	Yes	0.2. × 0.2 × 2.8	7:42
coronal	proton-densitiy turbo spin echo	2770 / 25	No	0.2. × 0.2 × 3.0	5:26
transverse	proton-densitiy turbo spin echo	6230 / 41	Yes	0.2. × 0.2 × 2.5	4:29

* Example represents the current standard MR imaging protocol for a 3 T MR unit (Skyra Siemens Healthcare, Erlangen, Germany) at the author’s institution using a dedicated 18ch-transmit-receive knee coil

Although MRI examination of the knee is relatively fast, it is not uncommon for the protocols to be “optimized” in clinical practice to shorten the acquisition time even more. However, technical improvements should be used to increase image quality and diagnosis accuracy, rather than to shorten the protocols.

### Examination planes

**Fig. 4:**
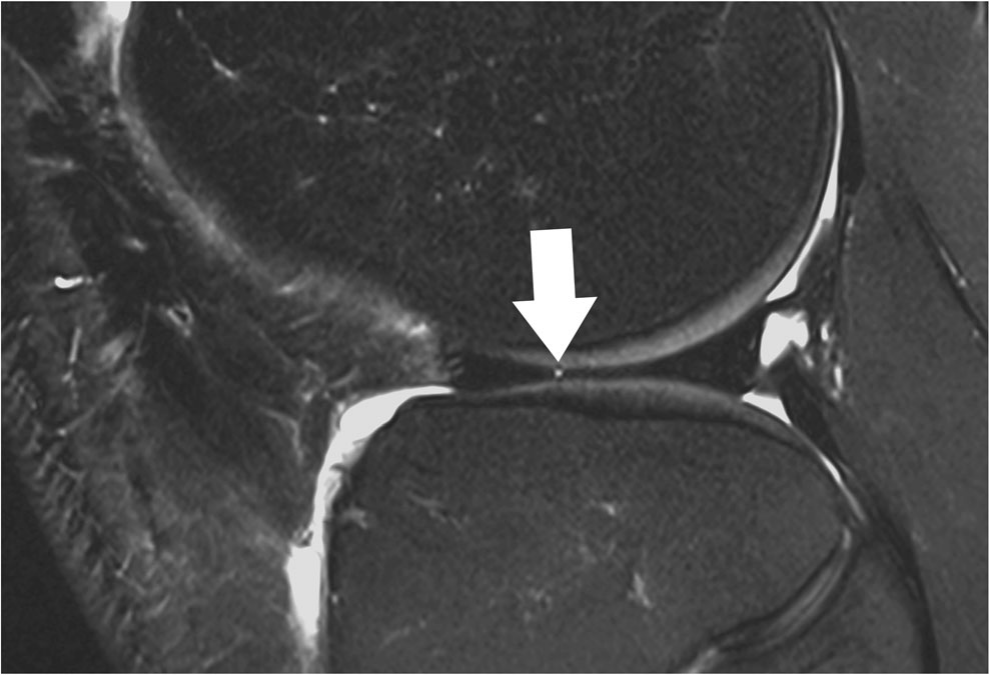
Sagittal T2-weighted, fat-suppressed fast spin echo image of the right knee in a 39-year-old male patient acquired at 3 Tesla (Siemens Healthcare, Erlangen, Germany) using a dedicated 28 channel transmit-receive knee coil shows a small radial tear of the lateral meniscus (*arrow*)

**Fig. 5:**
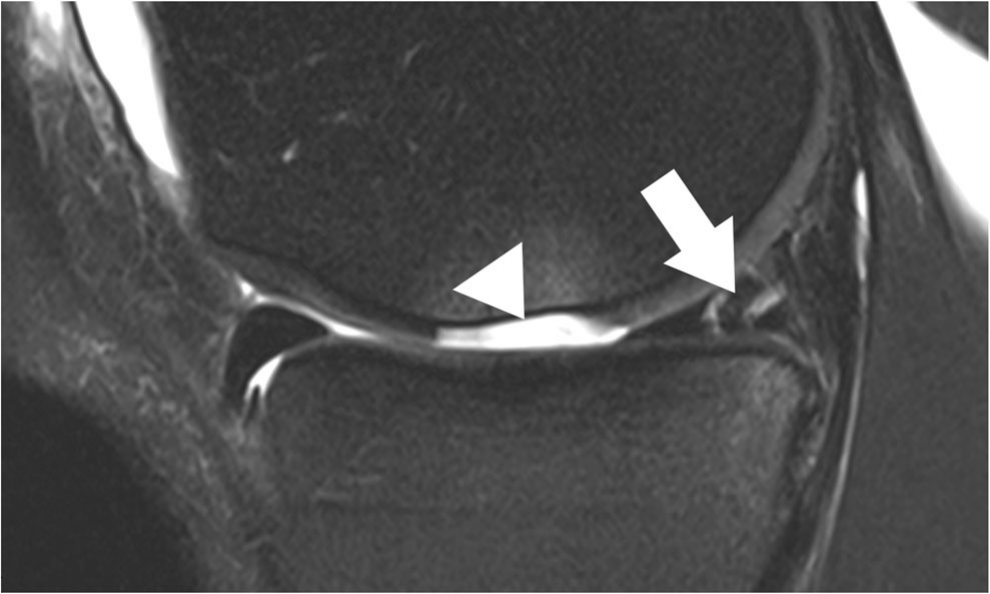
Sagittal T2-weighted, fat-suppressed fast spin echo image of the right knee in a 25-year-old male patient shows a complex tear (two parallel vertical longitudinal tears) of the posterior horn of the medial meniscus with three fragments (*arrow*), as well as a traumatic articular cartilage defect (*arrowhead*) with complete loss of an osteochondral fragment

Several meniscal lesions are best evaluated on sagittal images, including meniscal avulsion, radial tears (“ghost” meniscus sign), tears of the anterior horns, and posterior meniscocapsular separation (Figs. 4 and 5). Moreover, as many as 82 % of the meniscal tears are identified on sagittal images only [13]. The diagnosis mainly relies on the identification of an intrameniscal linear signal change and its contact with the meniscal surfaces. This includes that acquisition parameters are tailored such that potential meniscus tear can be detected, e.g. by having long echo time (TE) acquisitions for better fluid sensitivity. The number of the sagittal images in which the linear signal change contacts the meniscal surface may be an important factor in diagnostic accuracy and diagnostic confidence. The positive predictive value for diagnosing meniscal tears increases from 53 %, when the diagnosis is based on only one sagittal image in which the linear change extends to one of the meniscal surfaces, to 90 %, when the longitudinal linear signal contacts the meniscal surface in two consecutive sagittal images [5]. Therefore, in order to avoid false-positive reports, it is recommended that linear signal changes that contact the meniscal surface solely on one image are reported as possible tears [5, 10]. The false-positive diagnosis is even more likely in cases in which the intrameniscal signal contacts only the superior meniscal surface on sagittal images [5]. A correct evaluation of some particular types of tears (bucket-handle tears and radial tears) is difficult when it is based solely on sagittal images [13, 14]. In these cases, the images obtained in coronal and axial orientations play an important role.

**Fig. 6:**
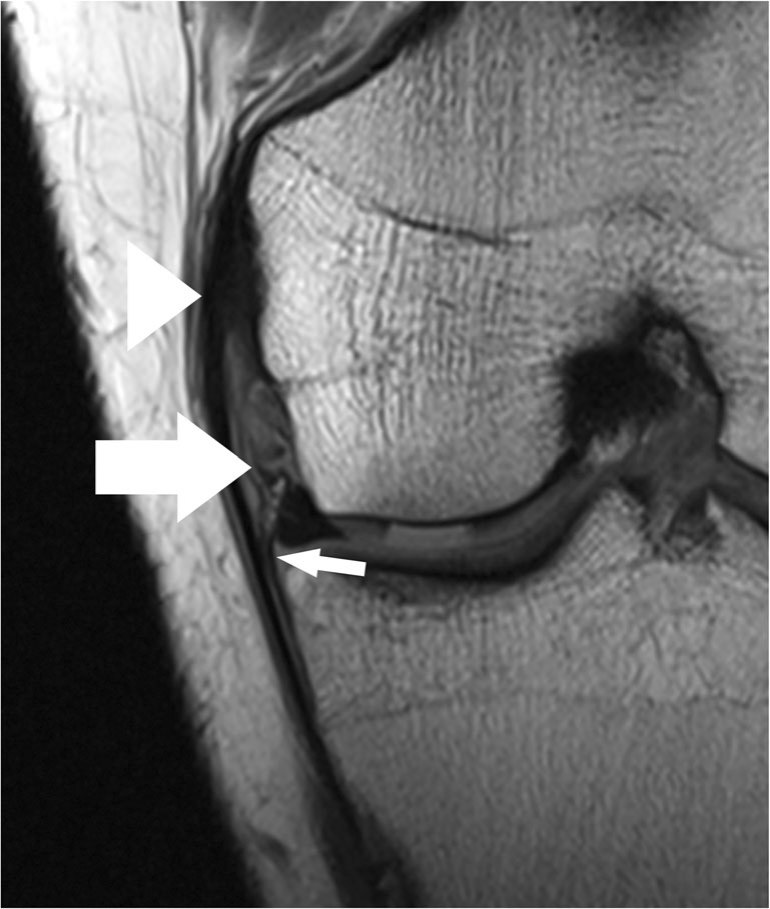
Coronal proton-density–weighted fast spin echo image of the right knee in a 25-year-old male patient with a traumatic osteochondral defect at the medial femoral condyle shows displacement of the medial meniscus and complete rupture of the meniscofemoral ligament (*arrow*). The more superficial medical collateral ligament (*arrowhead*) shows only mild abnormalities with thickening at its femoral insertion. Note the normal meniscotibial ligament (*small arrow*)

*Coronal images* provide valuable information regarding meniscal shape and its attachments. Some types of meniscal tears are better characterized on coronal images combined with sagittal images when compared to sagittal images alone [13]. Other types of meniscal tears (e.g. small radial tears) may be seen only in the coronal plane [13]. The horizontal tears that are seen on sagittal images may not always be accurately described with regard to the relation with the meniscal surface. In those cases, the evaluation of the coronal images allows a better evaluation of the lesion, especially when located in the body of the meniscus [13]. The coronal plane is particularly useful in diagnosing bucket-handle tears, detached meniscal fragments, and meniscal extrusions beyond the tibial plateau (Fig. 6). Meniscal root tears are also highly accurately diagnosed on coronal images [15].

**Fig. 7:**
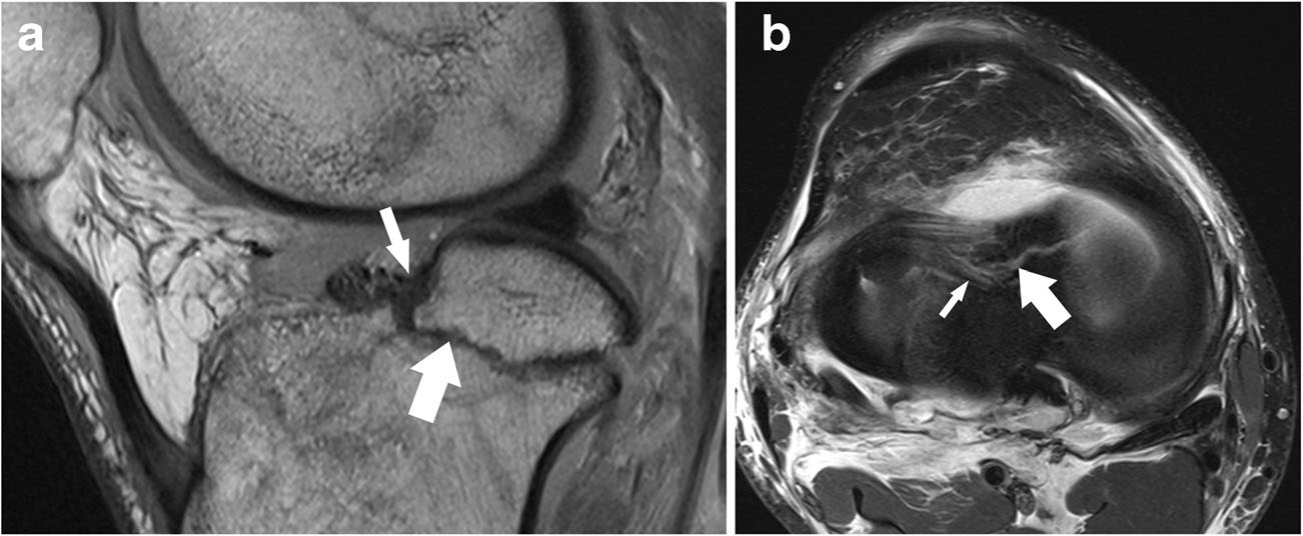
Sagittal proton-density–weighted fast spin echo image (**a**) of the right knee in a 25-year-old male patient with a severe knee trauma shows a fracture line (*arrow*) within the tibia and intrameniscal signal changes at the level of the anterior root (*small arrow*). Axial proton-density–weighted fat-suppressed fast spin echo image (**b**) shows the fracture line (*arrow*) within the tibia extending through the lateral meniscus’ anterior root, which is literally split into two halves (*small arrow*)

**Fig. 8:**
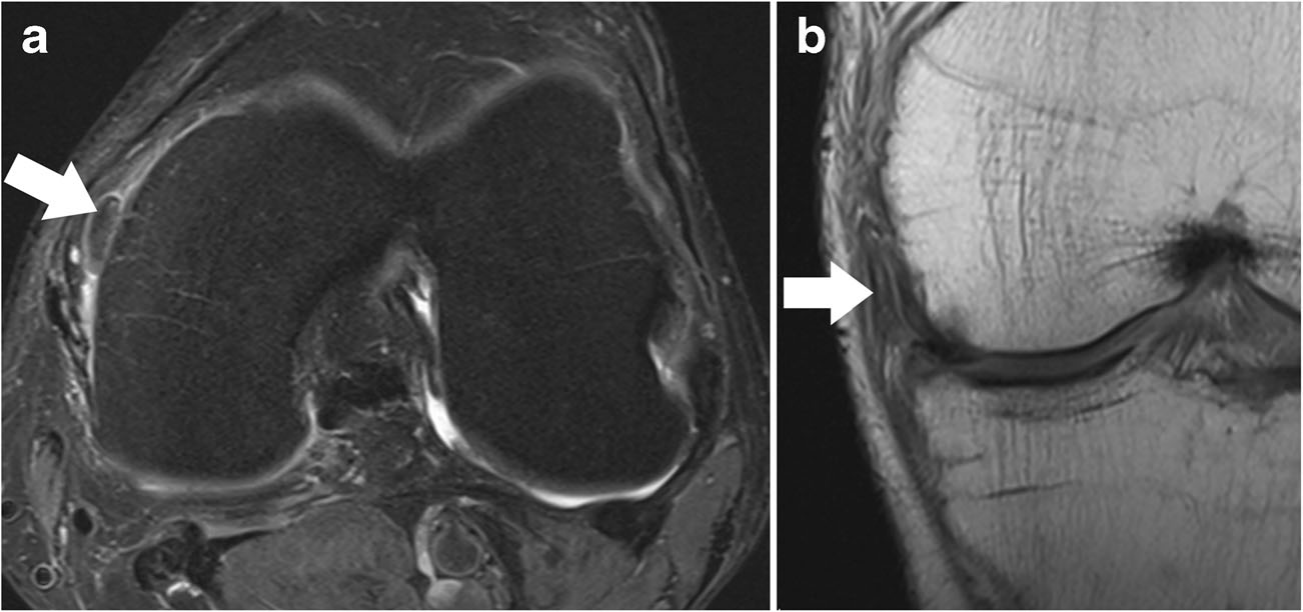
Axial proton-density–weighted fat-suppressed fast spin echo image (**a**) of the left knee in a 56 years old male patient shows an extra-articularly displaced fragment (*arrow*) of the medial meniscus. Coronal proton-density–weighted fast spin echo image (**b**) confirms the diagnosis and shows that the flapped portion (*arrow*) is still in continuity with the meniscus remnant (*arrow*)

*Axial images* are always part of clinical knee protocols and are routinely used for evaluation of the patellofemoral joint and ligaments. In the evaluation of menisci, it has been shown that diagnostic accuracy and diagnostic confidence of axial images are lower only when compared to sagittal and coronal images [16]. However, axial images are complementary to sagittal and coronal planes and may better show the whole extent of a meniscal tear (Fig. 7) [16]. It was shown that radial tears, complex tears (i.e. flap tears), and displaced fragments may be better visualized and localized in axial planes when high-resolution images are obtained (Fig. 8) [16–18].

### Pulse sequences

The routinely used MRI sequences are two-dimensional (2D), fast spin-echo (FSE) sequences (proton-density (PD)-weighted, T1—weighted sequences, and T2- and T1—weighted sequences with and without fat suppression). When comparing with spin-echo (SE) sequences, FSE sequences facilitate the examination in a shorter overall time without statistically significant differences in meniscal tear detection rate [19]. By using high-magnetic field strength with dedicated coils, and parallel imaging techniques, the scan time may be decreased even more. Some particular types of meniscal tears have been proven to be better evaluated on specific sequences (e.g. coronal T2-weighted images showed a 96 % diagnostic accuracy compared with 85 % accuracy of PD-weighted coronal images for diagnosis of medial meniscal roots tears) [15].

Most authors still prefer standard two-dimensional over three-dimensional fast spin echo sequences and over gradient echo sequences, because the former provide classic, well-known T1-, T2-, and intermediate-weighted contrast information and enable detection of subtle abnormalities of the meniscus and ligaments. This is in contradiction to the fact that with three-dimensional (3D) gradient-echo (GRE) and 3D fast spin-echo FSE sequences, thinner sections with decreased partial volume averaging and isotropic voxel imaging with multiplanar reformations (that potentially reduce the examination time) are possible. Indeed, several studies have shown that 3D isotropic FSE sequences can provide rapid knee joint evaluation. By using 3D fat-saturated PD FSE sequences at 3.0 T with isotropic resolution between 0.4 mm to 0.7 mm with acquisition times between 5 and 10 minutes, the authors found similar sensitivities and specificities as the routine MR protocol for detecting knee joint lesions [20–22]. However, the diagnostic performance of 3D sequences is not significantly increased in the overall evaluation of the meniscal tears [20, 23–25]. The exception might be the diagnosis of meniscal roots tears that can benefit from the better visualization of small structures by using 3D FSE images [24, 26]. Other authors considered that the image quality of the 3D sequences is lower compared with 2D FSE sequences, and the diagnostic confidence of the radiologists is still higher with the 2D sequences regarding the meniscal evaluation [24]. This difference might be the result of the decreased visualization of low contrast structures, such as meniscus, with the 3D sequences [24].

Recently, synthetic-echo time post-processing techniques for generating images with variable T2-weighted contrast demonstrated high sensitivity and specificity in evaluation of abnormalities of menisci [27]. Commercially available synthetic imaging techniques are currently being developed by some vendors, i.e. for brain imaging, and it is only a matter of time before we will see further clinical trials in the musculoskeletal system as well [27]. MR fingerprinting may also be a future development that could dramatically change the way we assess meniscus. Both quantitative and qualitative evaluation could be possible from only one acquisition, as well as reconstruction of images with different contrast information [28]. However, MR fingerprinting and synthetic imaging has yet to be given research status, although they will be available soon by at least one vendor. Currently, synthetic techniques for meniscal imaging should not yet be used routinely as there is only very little evidence.

Another important development that will soon likely find its way into clinical routine (3–5 years) is the acceleration of 2D sequences by multi-slice acquisition techniques. The potential reduction of acquisition time is in the range of twofold to threefold [29, 30]. Since the original sequences and their contrast remain unchanged, the acceptance of these new sequences and their adoption to clinical imaging protocols will likely be very high amongst musculoskeletal radiologists.

### Magnetic field strength

**Fig. 9:**
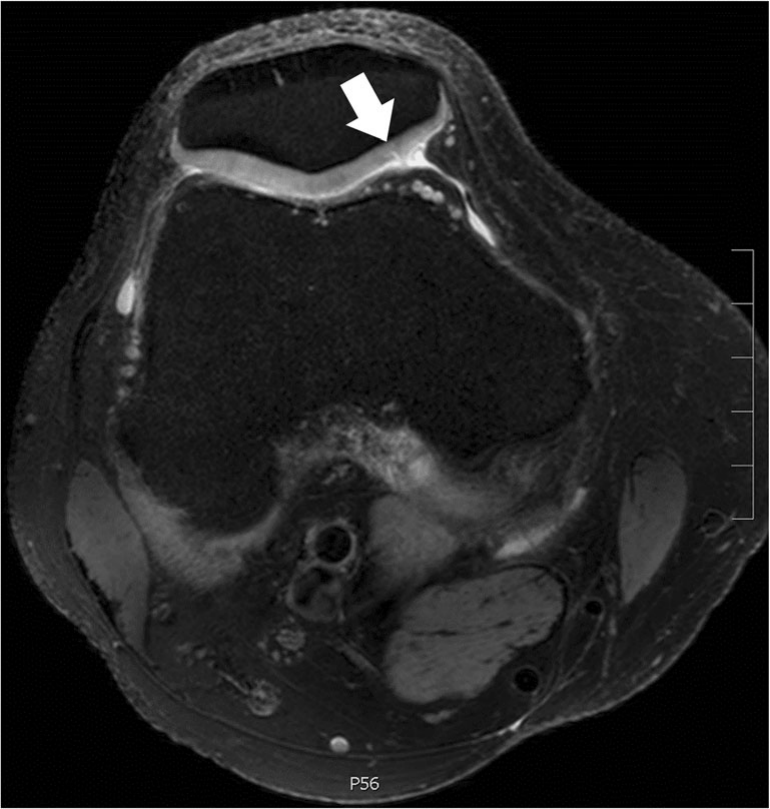
Axial proton-density–weighted fat-suppressed fast spin echo image of the right knee in a 40-year-old male patient acquired at 7 Tesla (Philips Healthcare, Best, the Netherlands) using a dedicated 28-channel transmit-receive knee coil shows a small articular cartilage fissure at the patella (*arrow*). Image in-plane resolution was 360 × 360 μm

Over the last decade, the expectations in terms of diagnosis accuracy has increased with the deployment of higher magnetic field strength. In many places, 3.0 T systems have replaced the 1.5 T equipment as standard of care. Musculoskeletal imaging is one of the fields that gains significantly from higher magnetic field strength when appropriate adaptation of pulse sequence parameters and high performance dedicated coils are used. The 3.0 T scanners have shown a significant improvement in visualization and evaluation of small structures such as ligaments, nerves, and articular cartilage compared to 1.5 T systems [31–33]. In addition, 4.0 T and 7.0 T MR units are increasingly available. Until now, more than 65 installed MR systems worldwide operating at 7.0 T have been established as platforms for clinically oriented research. The introduction of the 7.0 T scanner has pushed the possibilities of morphological, functional, metabolic and diffusion-weighted imaging of the tendons, cartilage, and trabecular bone significantly forward (Fig. 9) [34–37].

Although, there are data that show that higher magnetic field strength improves the diagnostic performance for anterior cruciate ligament (ACL) tears, cartilage and subchondral bone, the evidence regarding the added clinical value of higher fields MRI for meniscal tear evaluation is limited and is still a subject of debate [6]. Some authors consider that there is no significant difference for meniscal tear accuracy between magnetic field strengths ranging from 1.5 T to 7.0 T [6, 38–40]. However, as a result of an increased spatial resolution and increased signal-to-noise ratio, the detection and characterization of small tears (e.g. of the free margin of the meniscus), small radial tears, or tears of the meniscal roots are better assessed when higher magnetic fields are used. Therefore, we consider that higher magnetic field strengths may improve the diagnosis accuracy and diagnosis confidence, and we recommend that knee examinations at 3.0 T scanners should be used, at this moment, as the new standard in clinical practice.

### Typical pitfalls

Whereas most radiologists and clinicians are well aware of classic signs for meniscal tears (in either the anterior horn, body or posterior horn), typical pitfalls in the diagnosis involve the meniscal roots and inter-meniscal connections, as well as the ligamentous attachment of the medial and lateral meniscus. Knowledge of the normal anatomy is key for diagnosis. The subsequent paragraphs provide a comprehensive overview of typical pitfalls; classic signs are briefly reviewed.

### Meniscal roots and inter-meniscal connections

The anterior and posterior horns of the menisci are firmly attached to the tibial plateau through the insertional ligaments known as the meniscal root ligaments. They have an important role in knee biomechanics and kinematics by securing the meniscus in place and acting as anchors to the bone for both menisci [41]. Although meniscal root tears are less common than other types of meniscal tears, they can have a significant clinical impact. Cartilage defects and extrusion of the meniscus with respect to the tibial margin have been linked with tears of the meniscal roots [41–46]. There are three types of meniscal root lesions: avulsion injuries of the attachments, radial tears, and degenerative changes. Usually, they are located within few millimetres from the bony insertion [47]. An accurate diagnosis of such lesions as well as of associated injuries are mandatory for the treatment decision, i.e. nonoperative versus operative, in order to avoid a poor clinical outcome and long-term prognosis. Meniscal root tears are, in many cases, unrecognized and neglected in the MRI reports. In the literature, it is reported that, e.g. one-third of the tears of the posterior medial root are missed [48, 49]. Visualization of the meniscal roots on MRI is challenging, but increased awareness of the normal anatomy and the imaging signs of meniscal roots tears may improve the diagnosis accuracy (Table 2). It is important to keep in mind that the anatomy of the meniscal roots is not uniform and that there are slight differences in terms of the exact attachment site as well as MR appearance.

**Table 2:** Tibial insertions of the meniscal roots and meniscal roots tear MRI findings [41, 50–54]

Meniscal root ligament	Tibial insertion relative to cruciate ligaments	Meniscal root tear – MRI findings	
		Direct findings	Associated findings
Anterior medial root	7 mm anterior to the anterior cruciate ligament (ACL)	Ghost meniscal sign on sagittal images	Cartilage defect
Posterior medial root	8 mm anterior to the most superior insertion of the posterior cruciate ligament (PCL)	Linear defect on coronal images (truncation sign)	Medial or lateral meniscal extrusion ^1^
Anterior lateral root	4.1 mm lateral to the posterolateral bundle of ACL	Radial linear defect on axial images	Tear of ACL
Posterior lateral root	10.8 mm posterior to ACL and 12.7 mm anterior to PCL		

^1^ Meniscus is displaced beyond the tibial margin—on coronal images, a distance more than 3 mm for the medial meniscus and more than 1 mm for the lateral meniscus is considered abnormal [47]

#### Medial meniscal roots

**Fig. 10:**
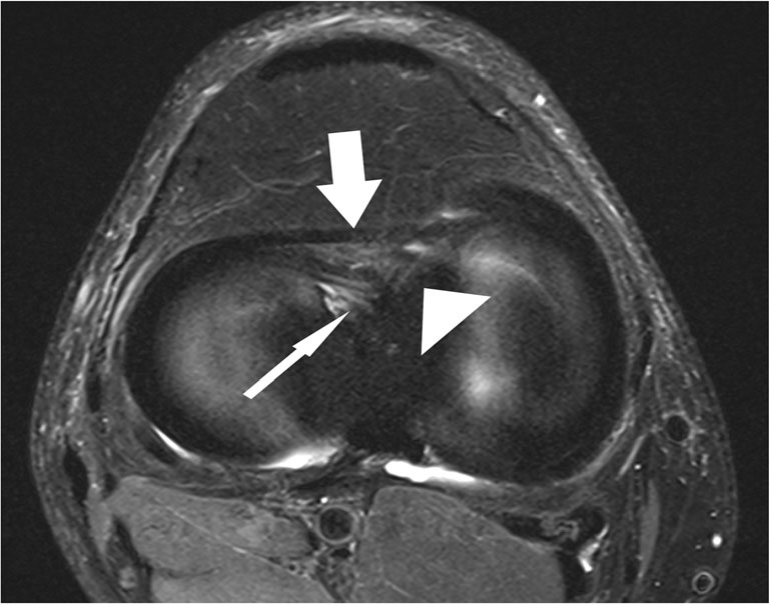
Axial proton-density–weighted fat-suppressed fast spin echo image in a 51-year-old female patient shows a (relatively thick) normal intermeniscal ligament (*arrow*) and normal anterior root of the medial meniscus (*small arrow*) and of the lateral meniscus (*arrowhead*)

The tibial attachment of the anterior horn of the medial meniscus or the anterior medial root ligament is situated at the intercondylar fossa, anteriorly to the anterior cruciate ligament (ACL) attachment (Fig. 10) (Table 2) [50]. In 59 % of the cases, the anterior horn of the medial meniscus is attached to the anterior cruciate ligament (ACL) [51]. The insertion area of the anterior medial root is the largest of any of the meniscus attachments [50–55]. Anatomically, four types of insertions have been described, but an accurate classification of these variants based on MR images is hardly possible and of no clinical relevance [56].

**Fig. 11:**
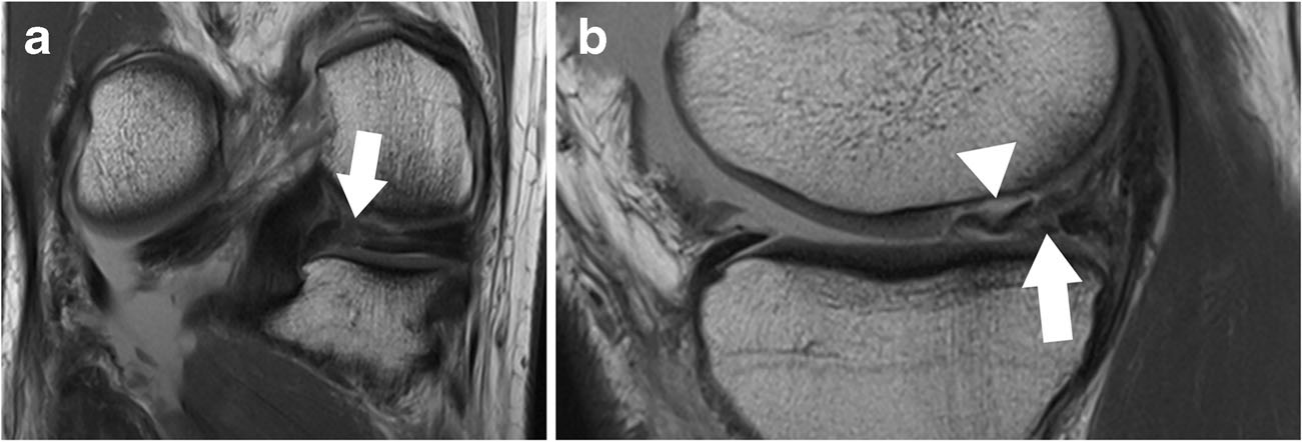
Coronal proton-density–weighted fast spin echo image (**a**) of the right knee in a 36-year-old male patient with a severe knee trauma shows a complex meniscocapsular injury, affecting both the posterior root of the medial meniscus and the posterior horn (*arrow*). Sagittal proton-density–weighted fast spin echo image (**b**) shows multiple meniscal fragments (*arrow*) some of which are displaced into a traumatic osteochondral defect (*arrowhead*)

The posterior medial root ligament attaches the posterior horn of the medial meniscus with the posterior intercondylar tibial fossa. The attachment site is situated between the insertion of the posterior lateral root ligament and the insertion of the posterior cruciate ligament (PCL) (Table 2) [57]. The posterior medial root ligament has the least mobility of all meniscus roots ligaments, and this may be the cause of the highest incidence of injuries compared with the other roots (Fig. 11) [58, 59]. Thus, after knee trauma, the posterior medial root ligament should be evaluated with extra care. Tears of medial meniscal roots are often associated with degenerative meniscal disease [60].

#### Lateral meniscal roots

**Fig. 12:**
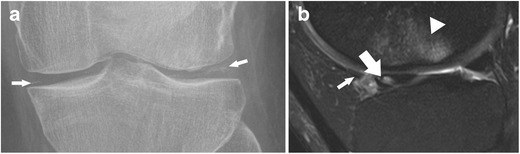
Standard radiograph (**a**) of the right knee in a 42-year-old male patient with a mild knee trauma shows typical signs of chondrocalcinosis with calcification (*small arrows*) within the articular space. Sagittal T2 weighted fat-suppressed fast spin echo image (**b**) shows traumatic bone marrow abnormalities (*arrowhead*) within the lateral femoral condyle and an oblique tear of the root of the anterior horn of the lateral meniscus (*arrow*). The tear should not be misinterpreted as the anterior transverse ligament or the geniculate ligament (*small arrow*), which is relatively thin, but intact in this patient

The anterior lateral meniscal root ligament attaches the anterior horn of the lateral meniscus to the lateral intercondylar tibial eminence just behind the anterior cruciate ligament (ACL) insertion (Figs. 10 and 12) (Table 2) [57]. It has been demonstrated that the anterior horn of the lateral meniscus is also attached to the anterior cruciate ligament (ACL) [51]. The posterior root ligament of the lateral meniscus attaches posterior to the lateral intercondylar tibial eminence and anterior to the medial posterior root ligament (Table 2) [57]. Posterior lateral root tear is highly associated with tear of the anterior cruciate ligament (ACL) [60].

#### Inter-meniscal ligaments

**Fig. 13:**
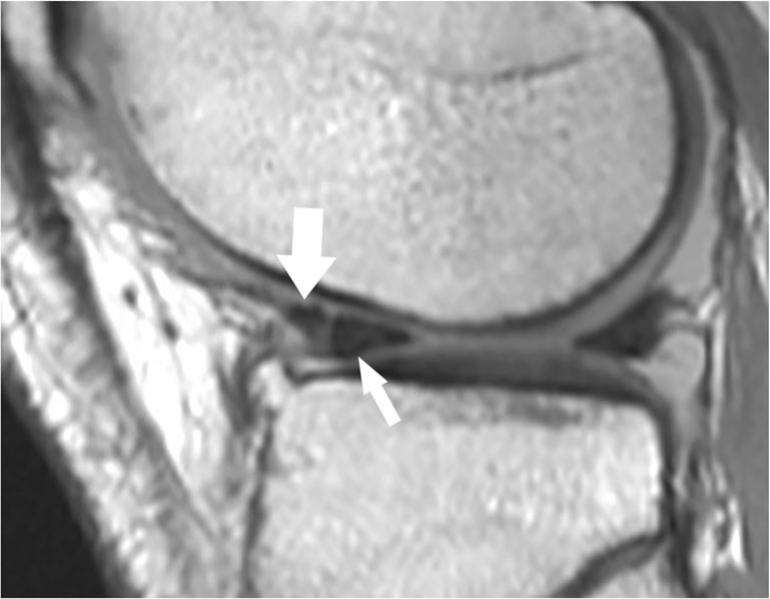
Sagittal proton-density–weighted fast spin echo image of the knee in a 74-year-old male patient with knee pain shows the normal anterior transverse ligament (*large arrow*) clearly separated by the normal anterior horn of the lateral meniscus (*small arrow*). The delineation between the two structures should not be misinterpreted as a vertical tear

The anterior transverse ligament, or the geniculate ligament, connects the anterior horns of the medial and lateral meniscus (Fig. 10). Medially, the transverse ligament blends with the posterior attachment of the anterior medial root ligament [56]. The ligament is inconsistently present, but, when present, there is an association between the transverse ligament attachment and the presence of tears in the medial meniscus as a result of restricting effect on anterior-posterior excursion of the anterior horn of the medial meniscus at lower degrees of knee flexion [61, 62]. The best planes for visualization of the ligament are sagittal and coronal [63]. On sagittal images, the presence of the geniculate ligament may lead to false-positive diagnosis of vertical longitudinal tear of the anterior horn or of displaced meniscal fragments (Fig. 13). The posterior transverse ligament is present much more rarely than the anterior transverse ligament [64]. It connects the posterior horns of the medial and lateral meniscus. It is seen on MR images, when present, on the coronal plane in front of the posterior cruciate ligament. Inconsistently, two oblique meniscomeniscal ligaments may be recognized on MR images [65]. The oblique ligaments extend from the anterior horn of the medial meniscus to the posterior horn of the lateral meniscus (medial oblique ligament) and from the anterior horn of the lateral meniscus to the posterior horn of the medial meniscus (lateral oblique ligament) [65].

### Capsular attachments

#### Medial meniscus

**Fig. 14:**
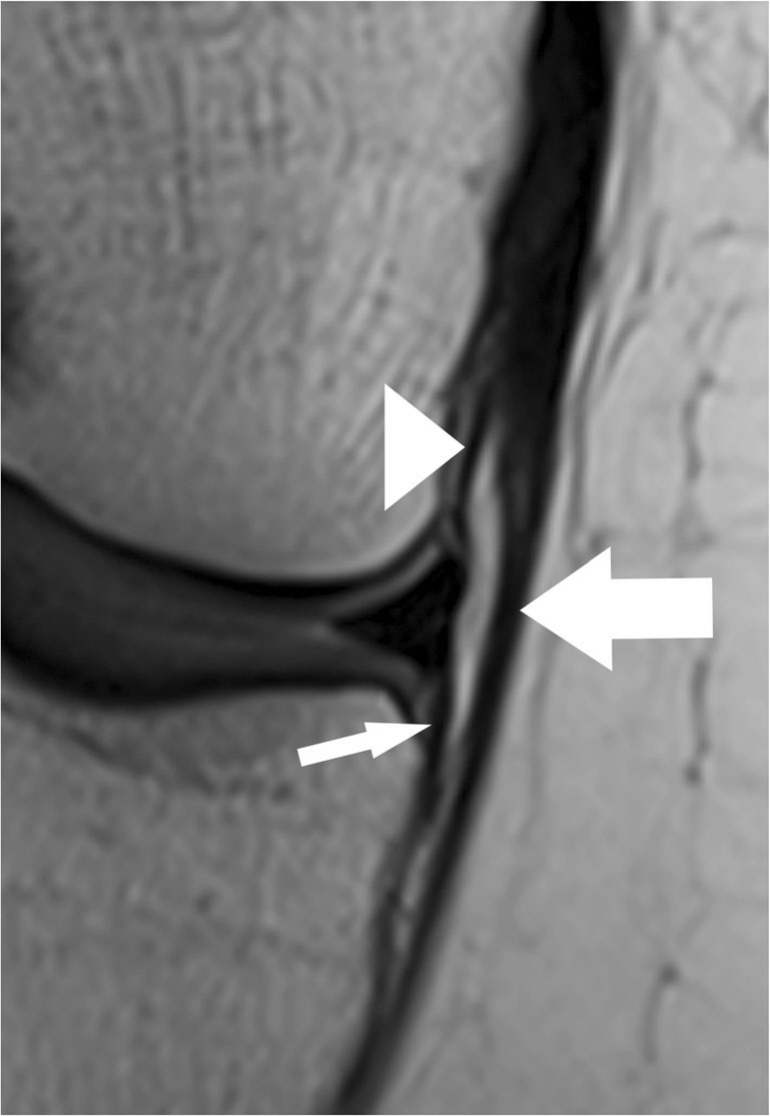
Coronal proton-density–weighted fast spin echo image in a 20-year-old female patient shows normal meniscotibial (*small arrow*), meniscofemoral (*arrowhead*) and medial collateral ligaments (*large arrow*)

**Fig. 15:**
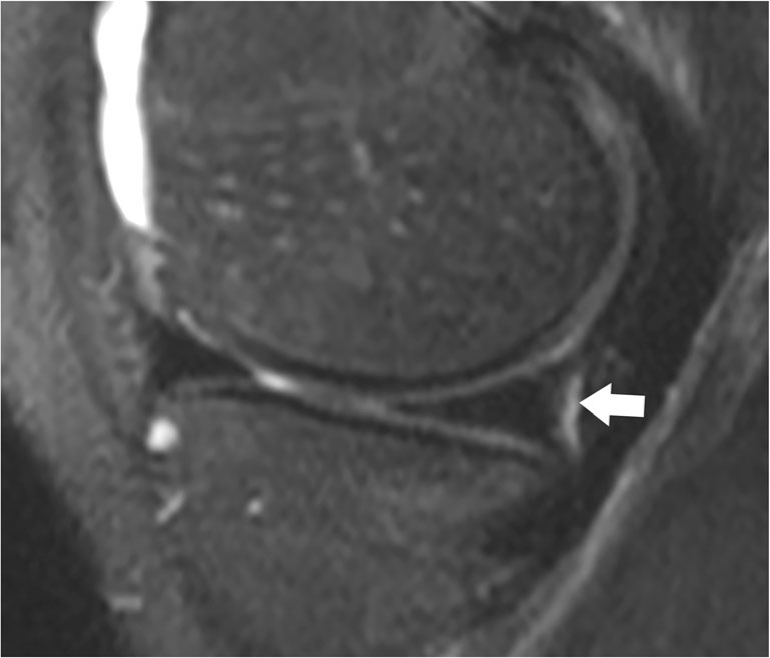
Coronal T2-weighted fat-suppressed fast spin echo image in a 21-year-old male patient shows the normal medial posterior femoral recess or medial gastrocnemius bursa with a small amount of fluid (*arrow*), which should not be diagnosed as a meniscocapsular separation

The attachment of the medial meniscus to the capsule is complex, and tears at this level are not always recognized and/or correctly described. The medial meniscus is closely attached to the knee capsule along its entire circumference [64]. The meniscofemoral, the meniscotibial, and the meniscopatellar ligaments are the structures that define the deep layer (layer 3) of the medial collateral ligament (Fig. 14) [64]. The meniscofemoral ligament is seen on MR images as a thin band that originates from the superior margin of the body of the medial meniscus and inserts on the femoral condyle 1–2 cm above the joint line [66]. The meniscotibial ligament is shorter and connects the inferior margin of the medial meniscus to the tibial cortex inferior to the joint line [66]. The meniscotibial ligament extends along the entire circumference of the posteromedial edge of the meniscus and further forms the deepest layer of the capsule. It is also called the coronary ligament or the meniscocapsular ligament [64]. The patellomeniscal ligament is seen on MR images anteriorly from the medial meniscus to the patellar margin [67]. A small bursa, known as medial posterior femoral recess or medial gastrocnemius bursa, separates the posterior horn of the medial meniscus from the joint capsule [68]. The presence of a small amount of fluid should not be misinterpreted as a meniscocapsular separation (Fig. 15).

Coronal MR images are particularly useful in demonstrating tears of the meniscofemoral and meniscotibial ligaments (Fig. 14) [64]. It should be noted, that tears of these ligaments can occur in isolation, with the more superficial layer of the medial collateral ligament appearing normal or slightly abnormal at the same time (Fig. 6). Nevertheless, it is important to recognize lesions of these attachments, as they stabilize the medial meniscus.

The presence of fluid with an increased distance between the medial collateral ligament and the medial meniscus, the displacement of meniscus from the tibia, a tear within the peripheral zone of the medial meniscus, and irregular meniscal margins are the best predictors of medial meniscocapsular separation (Fig. 11) [69]. The key sign for meniscocapsular separation is the presence of an abnormal signal intensity between the meniscus and the capsule or within the peripheral zone of the meniscus [64].

#### Lateral meniscus

**Fig. 16:**
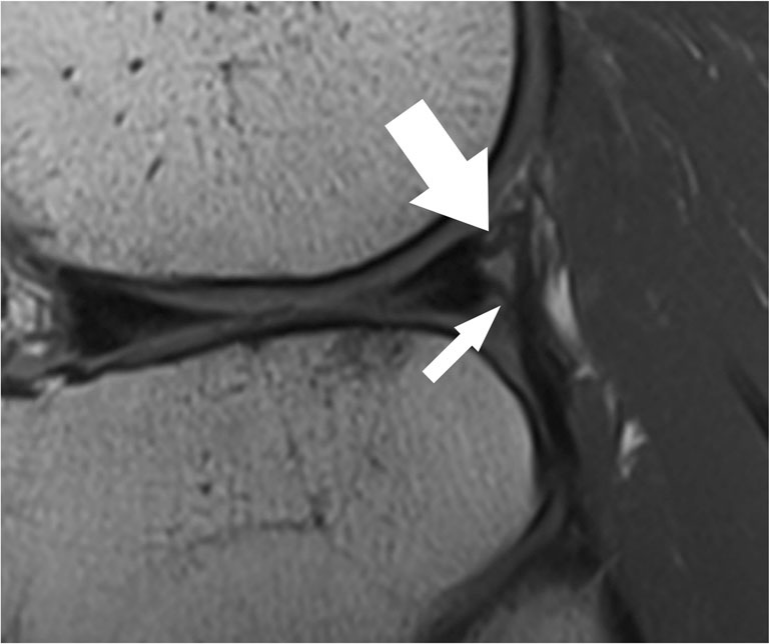
Sagittal proton-density–weighted fast spin echo image in a 40-year-old female patient shows the normal popliteomeniscal fascicles (PMF), which include the posterosuperior (*arrow*) and the anteroinferior fascicles (*small arrow*)

Unlike the medial meniscus, there is no attachment of the lateral meniscus to the lateral collateral ligament [64]. The lateral meniscus is attached to the tibia along its entire circumference through the lateral meniscotibial ligament, also known as the coronary ligament or the meniscocapsular ligament. The lateral meniscus is also strongly attached to the popliteus tendon and to the medial femoral condyle. Knowledge of the anatomy of these attachments is important in order to avoid reporting false positive diagnoses of posterior horn meniscal tears or free meniscal fragments. The popliteomeniscal fascicles (PMF) are part of the posterolateral corner of the knee and connect the posterior horn of the lateral meniscus with the popliteal tendon (Fig. 16). The posterosuperior popliteomeniscal fascicles extend from the posterolateral aspect of the lateral meniscus to the popliteus tendon, and the anteroinferior popliteomeniscal fascicles extend from the middle third of the lateral meniscus to the popliteus tendon [70]. The anteroinferior popliteomeniscal fascicles are stronger and shorter than the posterosuperior popliteomeniscal fascicles [71, 72]. On MR images, they are inconsistently seen as hypointense structures on sagittal planes (Fig. 16) [70]. A displaced lateral meniscus and the disruption of the popliteomeniscal fascicles with high-signal intensity soft tissue oedema with or without perimeniscal fluid are suggestive of a possible lateral meniscocapsular separation [73].

**Fig. 17:**
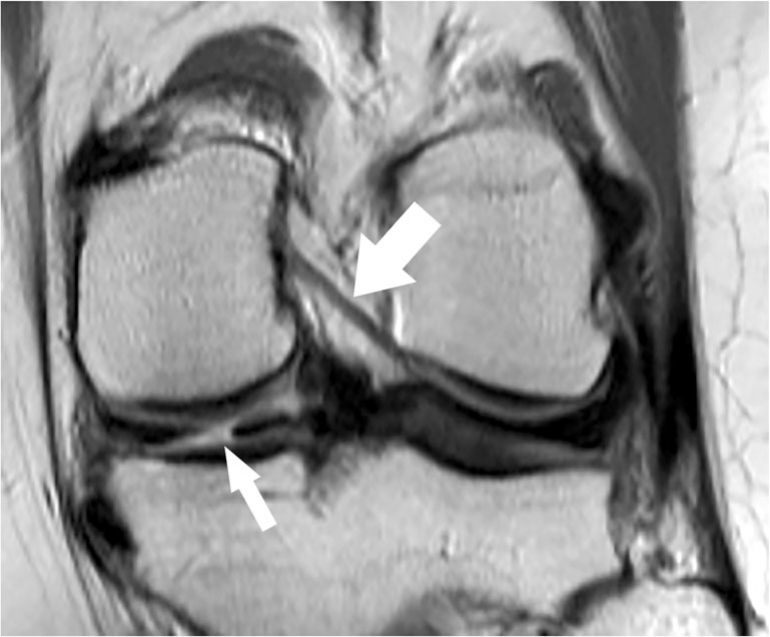
Coronal proton-density–weighted fast spin echo image in a 47-year-old female patient shows the normal posterior meniscofemoral ligament (also referred to as Wrisberg ligament) extending from the posterior horn of the lateral meniscus to the medial femoral condyle (*large arrow*). Note a radial tear of the posterior horn of the medial meniscus (*small arrow*)

**Fig. 18:**
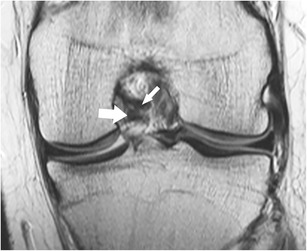
Coronal proton-density–weighted fast spin echo image in a 19-year-old male patient shows the normal anterior meniscofemoral ligament (Humphrey ligament) (*large arrow*) inferior to the posterior cruciate ligament (*small arrow*)

**Fig. 19:**
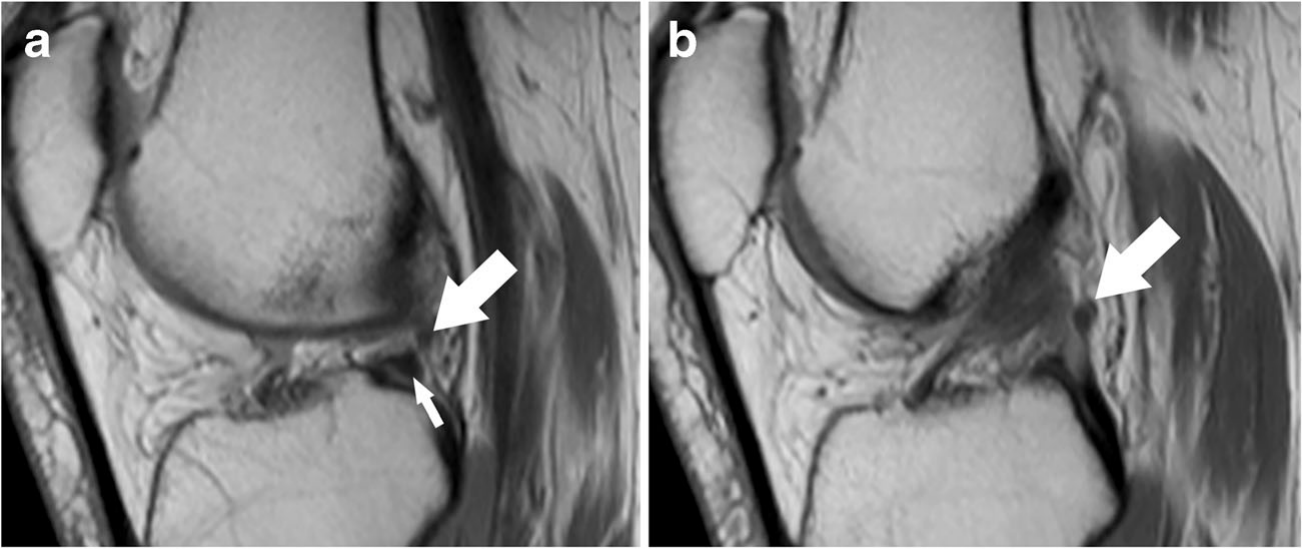
Sagittal proton-density–weighted fast spin echo images in a 43-year-old female patient through the lateral meniscus. At the insertion of the posterior meniscofemoral ligament (**a**), the ligament (*large arrow*) closely parallels the outer posterior margin of the posterior horn of the lateral meniscus (*small arrow*). The linear signal between the low-intensity meniscus and the low-intensity ligament may lead to pitfalls and could be confused with a detached meniscal fragment. However, on the next more medial sagittal image (**b**), the ligament (*large arrow*) is clearly visible posteriorly to the posterior cruciate ligament

The posterior horn of the lateral meniscus attaches to the medial femoral condyle through the commonly well-known *meniscofemoral ligaments*. The posterior meniscofemoral ligament (also referred to as Wrisberg ligament) extends from the posterior horn of the meniscus to the medial femoral condyle proximal to the posterior cruciate ligament (PCL) insertion (Fig. 17). The anterior meniscofemoral ligament (also referred to as Humphrey ligament) attaches on the medial femoral condyle, inferior to the posterior cruciate ligament insertion (Fig. 18). Many variations of these ligaments have been reported, and the incidence of the presence of one or the other ligament is 70–100 % [74]. At their meniscal origin, the ligaments closely parallel the outer posterior margin of the meniscus, and, on sagittal images, the linear signal between the low-intensity meniscus and the low-intensity ligaments may lead to pitfalls and could be confused with a tear (Fig. 19).

Isolated lesions of the ligament of Humphrey or Wrisberg are rare. Most often, the ligaments are affected by tears of the posterior horn when both ligaments may show a wavy course. On the other hand, isolated distortions of the ligaments are an indirect sign for lateral meniscal root tears.

### Patterns and classification of meniscal tears

An accurate description of meniscal tears is of utmost importance. A comprehensive report includes the exact localization, orientation, and extension of the meniscal tear. For the latter, it should always be noted to which surface the tear extends to (femoral surface or tibial surface). A clear description may obviate unnecessary surgery or may enable a better surgical planning.

**Fig. 20:**
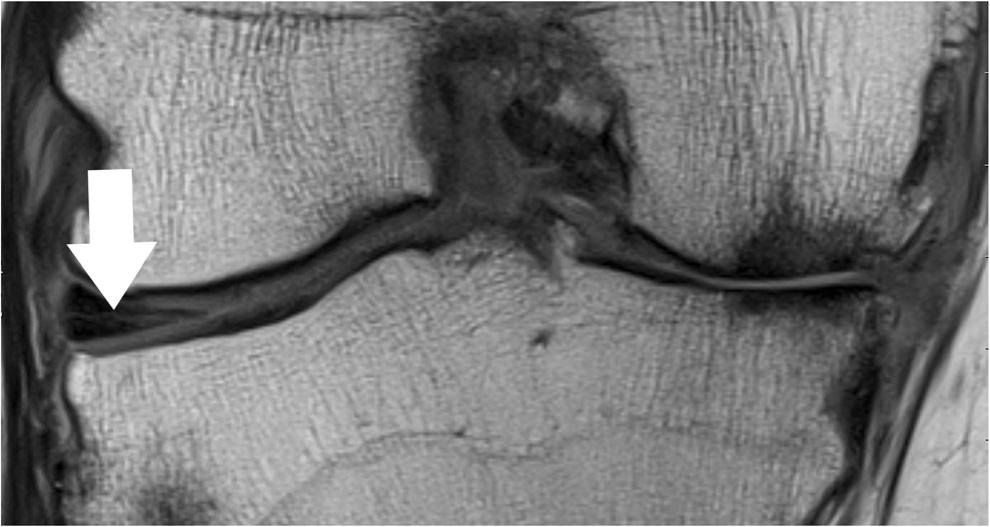
Coronal proton-density–weighted fast spin echo image of the right knee in a 61-year-old male patient with severe osteoarthritis shows a chronic (stable) horizontal tear (*arrow*) of the lateral meniscus

**Fig. 21:**
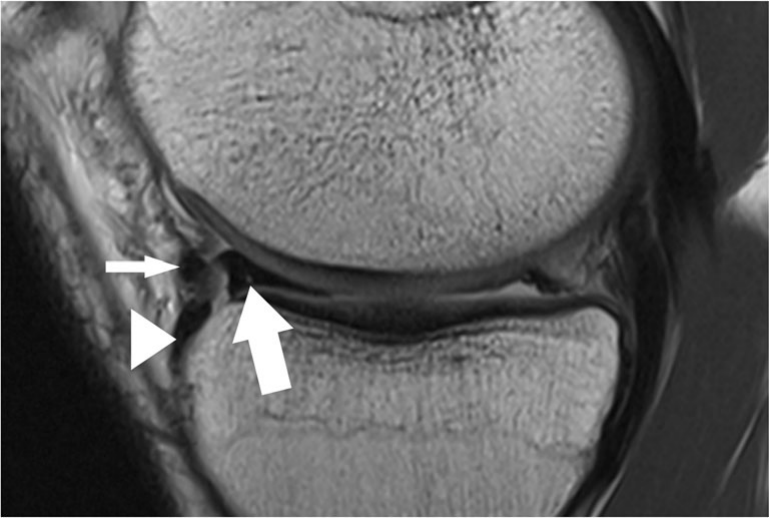
Sagittal proton-density–weighted fast spin echo image of the left knee in a 22-year-old male patient with a bucket handle tear of the medial meniscus shows the displaced anterior horn (*arrow*) which lies posterior to its root insertion (*arrowhead*). Note the relatively thick intermeniscal ligament (*small arrow*) that normally connects both anterior horns of the menisci as well as the small non-displaced remnant of the posterior horn

**Fig. 22:**
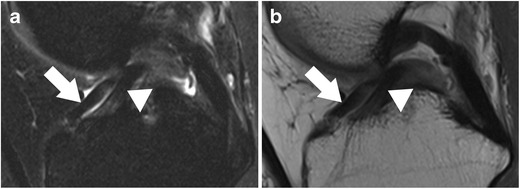
Sagittal T2 weighted fat-suppressed (**a**) and corresponding proton-density (PD) weighted fast spin echo image (**b**) of the right knee in a 73-year-old female patient with a bucket handle tear show the classic “double anterior cruciate ligament sign” that is formed by the displaced meniscus (*arrow*) that lies anterior to the anterior cruciate ligament (*arrowhead*)

The traditional description of a meniscal tear is that of a linear increased signal intensity within the meniscus. According to the orientation, a meniscal tear can be vertical, horizontal, or complex (Table 3). An horizontal tear is defined as a linear signal abnormality involving the surface of the meniscus in a horizontal orientation of less than 30° relative to the adjacent tibial plateau (Fig. 20) [5]. Vertical tears are subdivided into radial and longitudinal tears. Radial tears are perpendicular to the long axis of the meniscus and begin in the free edge of the meniscus (Fig. 4) [5, 75]. Vertical longitudinal tears are parallel to the long axis of the meniscus, away from the free edge (Fig. 5) [5, 75]. A complex tear refers to a combination of more orientations (e.g. parrot beak tear). Tears with displaced fragments such as bucket-handle tear, flap meniscus tear, or free meniscus fragment are also classified as complex tears (Figs. 21 and 22).

**Table 3:** Meniscal tear classification [1, 64, 75–78]

Meniscal tear	Vertical	Longitudinal	Incomplete (linear signal changes only involving one meniscal surface)	Stable	Low clinical relevance, not always correlated with symptoms = “leave alone” lesion
			Complete (linear signal changes involve both meniscal surface)	Unstable when meniscocapsular separation is present or in extensive lesion	High clinical relevance
		Radial	Without meniscal root involvement	Usually unstable	High clinical relevance
			Radial meniscal root tear		
	Horizontal or oblique-horizontal	Incomplete (linear signal changes not involving a meniscal surface)		Stable	Low clinical relevance, not always correlated with symptoms = “leave alone” lesion
		Complete (linear signal changes involving one or more meniscal surfaces)		Unstable/stable	Different clinical relevance depending on associated lesions, e.g. anterior cruciate ligament (ACL) tear, medial collateral ligament (MCL) tear
	Complex	Horizontal and vertical tear, complex linear pattern		Unstable	High clinical relevance
		Bucket-handle			
		Flap tears (including parrot beak tears)			
		Free meniscal fragment

Meniscal grading, e.g. grade I, II, or III, which has been widely used in the past, is now considered obsolete in clinical routine imaging [79]. As with many classifications in musculoskeletal imaging, multiple versions of the same classification system were used, mixed up, or inconsistently used. This often caused misunderstanding, i.e. the referring physician could not distinguish between important and non-important lesions. Thus, a standard knee report may better distinguish between stable and unstable tears (Table 3), if there is a need for a classification system at all. Therefore, the orientation, the extension and involvement of meniscal surfaces (none, one, or both) are important features that should be recognized and described in the report. Stable tears (Fig. 20) have a potential for healing conservatively, whereas unstable tears often require surgery [1, 76]. It should be noted that the natural healing of meniscal tears might cause problems in the evaluation of follow-up MR images. Spontaneous healing is often associated with the presence of haemorrhage around the meniscal lesion [80]. Meniscal healing should not be misinterpreted as early re-tear.

The evaluation of post-operative meniscus can be challenging, e.g. when information about the type of prior surgery is not provided. The type of surgery defines the post-operative meniscus appearance. The two typical surgical approaches are meniscus preserving versus non-preserving therapies. The former includes meniscal suture, glueing, needle trephination or synovial abrasion. Non-preserving therapies include partial or total meniscectomy. Accordingly, normal intrasubstance changes in the operated meniscus after preservation surgery must be differentiated from a re-tear, which is usually characterized by a linear abnormal signal intensity that is more hyperintense than seen in healing meniscus. Intra-articular contrast application may help to differentiate re-tears (where the contrast is leaking into the cleft) from healing (where granulation and scar tissue fills up the cleft and where the contrast has no space to leak in). Missing parts of the meniscus may disclose non-preserving therapy. Sharp or truncated meniscal edges and loss of substance indicate meniscectomy.

A special category is the surgical repair of root tears. Usually, they challenge the surgeon in that the meniscus must be re-attached to bone. Surgeons may use arthroscopically assisted bone suture anchors (all-inside technique) or an intraosseous suture technique (“pullout technique”) [81]. To avoid misinterpretation of post-operative MR images, details about the type of surgery and the procedure are mandatory.

## Conclusion

Magnetic resonance imaging represents a standard tool in knee evaluation with a high specificity and sensitivity in diagnosing meniscal tears. However, in order to avoid errors of interpretations and pitfalls, there are several factors that should be taken into consideration. The technical platform and the sequence parameters, the awareness of the normal meniscal anatomy, and the knowledge of the patterns of the tears may influence the accuracy of diagnosis. An accurate and a complete description of meniscal tears is important and influences treatment planning.
